# Bi-Directional Fabry–Perot Cavity Antenna Based on Polarization-Dependent Transmit–Reflect Metasurface

**DOI:** 10.3390/s25216642

**Published:** 2025-10-30

**Authors:** Yanfei Ren, Zhenghu Xi, Tao Wang, Qinqin Liu, Shunli Zhang, Zhiwei Sun, Boyu Sima, Hao Zeng

**Affiliations:** 1 The 10th Research Institute of China Electronics Technology Group Corporation, Chengdu 250102, China; swjturen@163.com; 2 School of Electronic Science and Engineering, Nanjing University, Nanjing 210023, China; 3 Shanghai Radio Equipment Research Institute, Shanghai Academy of Spaceflight Technology, Shanghai 201109, China; xizhenghu@163.com; 4 Key Laboratory of Near-Range RF Sensing ICs and Microsystems (NJUST), Ministry of Education, School of Electronic and Optical Engineering, Nanjing University of Science and Technology, Nanjing 210094, China; 15253033693@163.com (T.W.); irene98van@163.com (Q.L.); smby@njust.edu.cn (B.S.); 5 School of Microelectronics and Communication Engineering, Chongqing University, Chongqing 400044, China; haoz@cqu.edu.cn

**Keywords:** Fabry–Perot cavity (FPC) antenna, bi-directional radiation, partially reflective metasurface (PRMS), low-profile

## Abstract

Metasurfaces (MSs) have been an effective method for the manipulation of electromagnetic (EM) radiation. However, this research mainly focused on controlling single-directional radiation. In this paper, a Fabry–Perot cavity (FPC) antenna based on the MSs technique is proposed, which obtains a bi-directional radiation with independent control of the forward and backward radiation patterns. The antenna is located in an FPC with two MSs forming the top and bottom surfaces. The MSs can partially reflect the *x*-polarized incident wave, i.e., it is a partially reflective metasurface (PRMS). Meanwhile, it can transform a specific incident component from *x*-polarization into *y*-polarization with a transmittance around −9.2 dB. In addition, the phase of the *x*-polarized reflection and *y*-polarized transmission can be controlled independently. So, a bi-directional radiation, of which the forward and backward radiation can be independently controlled, is obtained by the FPC antenna by manipulating the transmission phase distribution of the two PRMSs. As validation, two bi-directional radiation FPC antennas are designed based on the proposed method. Antenna 1 achieved a bi-directional single-beam radiation, of which the forward and backward radiation radiate to 2° and 177° with a gain of 13.4 dBi and 12.3 dBi, respectively. Antenna 2 achieved a bi-directional multibeam radiation, which radiates dual beams forward and a single beam backward. The two beams forward fire to 37° and 322° with a gain of 9.53 dBi and 9.3 dBi, while the beam backward fires to 178° with a gain of 7.8 dBi. At last, the first antenna is fabricated and measured for experimental validation, achieving the coincident results as simulation. This research can be potentially applied in research on antennas, communication, and wireless sensors in several practical scenarios, such as multibeam electromagnetic radiation, multi-user communication, multi-target monitoring, and sensor–communication system integration.

## 1. Introduction

Recently, the combined sensor–communication system has been a focused issue in sensor research, as it can transmit the perception information to the information processing system wirelessly and instantly. With abundant spectrum resources, high data transmission rates, and compact size, the millimeter-wave (mm-wave) band has been widely used in communication and sensor systems [1,2,3]. Conventional antenna designs face problems such as large volume, inflexible radiation directivity, and limited beam steering capability. The new Fabry–Perot cavity (FPC) antenna [4,5,6] method based on metasurface technology has become a hot research topic. One of its key functions is the convenient manipulation of bi-directional radiation [7]. The conventional method to realize bi-directional radiation is to place two antennas or antenna arrays back-to-back [8,9,10]. However, feed networks suffer from high complexity and high insertion loss, especially for the bi-directional antenna arrays [11,12]. Nowadays, FPC antennas, which can obtain bi-directional radiation in multiple spatial ranges with improved range coverage, have been widely applied in wireless communication systems. They utilize the metasurfaces (MSs) as the cavity surfaces, which can solve the problems in classical transmissive or reflective MS antenna designs, such as feed blockage [13] and a high profile [14]. FPC antennas obtain the precise manipulation of electromagnetic waves through artificially designed electromagnetic structures, especially in the areas of beam control, polarization conversion, and radiation pattern control.

In the MS-FPC antenna, both the transmittance and reflectance of the employed MS should be accurately designed. This can refer to other antenna research, the transmit–reflect array (TRA), which can achieve bi-directional radiation with high efficiency and design flexibility. There are some conventional methods for achieving bi-directional high-gain beams, for example, (i) by cascading phase-shifting surfaces and frequency-selective surfaces (FSS), the phase-shifting surfaces can operate in transmission and reflection modes in different wavelength bands to generate bi-directional beams [15,16]; (ii) a symmetric bi-directional beam can be achieved by using a cross-polarized field from a single-layer metallic structure [17,18]. The first method requires the antenna to operate in two separate frequency bands [19,20]. To enable bi-directional radiation to operate at a single frequency, orthogonal polarization states were used [21,22]. However, the distance between the feed antenna and the TRA plane is usually more than a few times the wavelength, resulting in a high profile. This disadvantage can be solved by introducing the FPC, since the feeding wave undergoes multiple reflections and transmissions within the cavity [23,24]. The profile of the MS-FPC antenna is significantly reduced, with some other advantages such as a simplified feed network configuration, low cost, and facile fabrication [25,26].

This work presents an FPC antenna with low-profile and bi-directional radiation by introducing partially reflective MSs with certain transmittance and reflectance at 11.5 GHz. Meanwhile, in the MSs, the phase and polarization of transmission components are manipulated to obtain independent control of the forward and backward beams. The MSs partially reflect the *x*-polarized wave and make its wavefront divergent, while transmitting the *x*-polarized wave into *x*-polarization with a certain magnitude. In this case, based on the orthogonality of cross polarizations, the *y*-polarized transmission can be independently manipulated without mutual effect to the *x*-polarized reflection. The introduction of polarization conversion can simplify the structure design and provide more functionality. The phase of the *y*-polarized transmission was 1-bit phase-controlled, while the phase of *x*-polarized reflection was kept, so that bi-directional radiation is achieved by FPC antenna, of which the forward and backward beams can be independently controlled in *y*-polarization. Two verification examples are designed and simulated, including a dual-beam radiation and a tri-beam radiation FPC antenna. At last, the dual-beam FPC antenna is fabricated and measured as an experimental verification [27,28]. Both the simulation and measurement present consistent results as theoretical prediction on the proposed method. This research presents a feasible method for the FPC antenna by introducing the manipulation of polarization features. It has a low profile and enhanced functionality, which can be applied in various research areas, such as wavefront beamforming, communication, radars, and wireless sensors.

## 2. Design Principle

**Figure 1:**
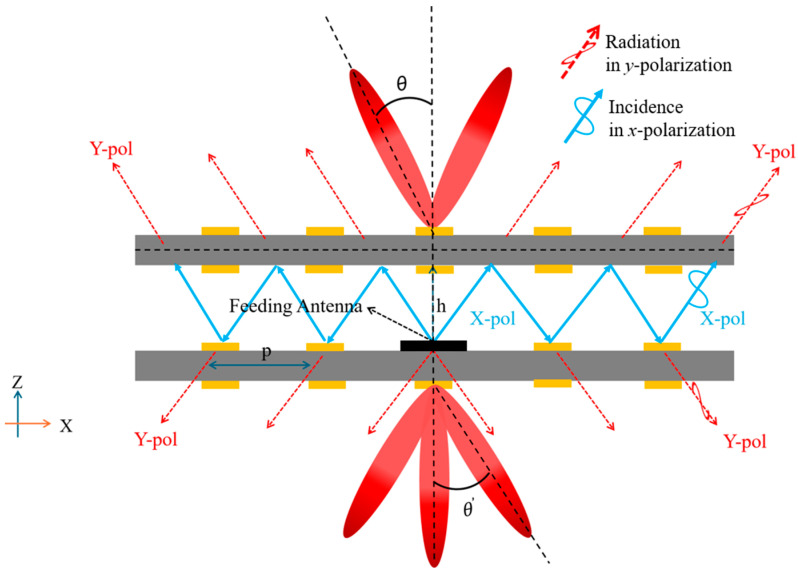
Schematic diagram of FPC antenna with independent bi-directional radiation method.

The schematic of the proposed bi-directional multibeam array antenna based on the combination of the Fabry–Perot (FP) antenna and metasurface technique is shown in Figure 1. The conventional FPC antenna mainly consists of a partially reflective surface (PRS), a feeding antenna, and a reflective ground. In the proposed bi-directional radiating FPC metasurface antenna scheme, the PRS in the conventional FPC antenna is replaced by a partially reflective metasurface (PRMS) with polarization conversion function. The reflective ground is also replaced by another PRMS. The proposed PRMS has proper transmittance and reflectivity, while its transmission-phase and reflection-phase distributions can be independently controlled. For bi-directional multibeam radiation, we herein control the transmission phase of the PRMS units and keep the reflection phase constant. The transmission-phase distribution on the two PRMSs is used to control the radiation direction, and the reflected phase is used to match the FPC antenna cavity height to ensure that the EM wave propagates between the top and bottom layers to satisfy the FP resonance criterion.

The FP resonance condition satisfies the following,

(1) $$-\frac{4\pi h}{\lambda }+\varphi^{\prime }+\varphi^{\prime \prime }=2\pi N,N=0,\pm 1,\pm 2,\ldots .$$

in which $\varphi^{\prime }$ denotes the reflection phase of the top PRMS layer, $\varphi^{\prime \prime }$ denotes the reflection phase of the bottom PRMS layer, and *h* is the cavity height.

Control of the multibeam radiation is achieved by adjusting the transmission-phase distribution of the PRMS, which may affect the radiation characteristics of the feed antenna. This is similar to the scattering pattern of MSs or the radiation pattern of phased antenna arrays. In the proposed MS-PFC antenna, when the distance between the top and bottom layers *h* and the reflection phases $\varphi^{\prime }$ and $\varphi^{\prime \prime }$ satisfy the resonance conditions, ray-tracing analysis shows that the electromagnetic wave incidents on the PRMS at different locations maintain roughly the same phase. This demonstrates that the incident phases of all the PRMS units are basically the same. In order to form the desired beam shape, the transmission-phase distribution needs to be specifically designed on the PRMS, which implies that the units need to be phase-compensated at different locations of the PRMS. Therefore, the transmission-phase compensation is the key technology for the multibeam MS antenna design. The accuracy of the phase compensation directly determines the radiation performance of the antenna. In order to quantitatively analyze the phase-compensation distribution of the bi-directional MS, the 2 × N array shown in Figure 1 is explored as an example. In this case, the PRMS unit can be regarded as an ideal point source, and, according to the point source array theory, the distribution for the forward radiation direction mapping can be expressed as

(2) $$F_{f}\left(\theta^{f}\right)=\sum_{n=1}^{N}\;A_{n}^{f}\cdot e^{-j\left(k_{0}\mathrm{npsin}\theta^{f}+\varphi_{n}^{f}\right)}$$

where $\theta^{f}$ denotes the angle between the direction of radiation and the normal direction (*z*-axis direction), and $k_{0}$ denotes the number of waves in free space. $A_{n}^{f}$ denotes the amplitude of the electric field radiated by the *n*th unit in the top PRMS, which is inversely proportional to the distance from the central feed to the metasurface unit. *P* denotes the distance between the centers of the neighboring units. $\varphi_{n}^{f}$ is the transmission phase compensated by the corresponding units.

In a similar manner, the expression for the radiation pattern in the backward direction can be expressed as

(3) $$F_{f}\left(\theta^{b}\right)=\sum_{n=1}^{N}\;A_{n}^{b}\cdot e^{-j\left(k_{0}\mathrm{npsin}\theta^{b}+\varphi_{n}^{b}\right)}$$

where $\theta^{b}$ denotes the angle between the direction of backward radiation and the negative normal direction (negative *z*-axis direction). $A_{n}^{b}$ denotes the amplitude of the electric field radiated by the *n*th unit of the bottom PRMS, which is inversely proportional to the distance from the central feed to the MS unit. $\varphi_{n}^{b}$ is the transmission-phase compensation applied to the corresponding units. Note that the assumption here is to neglect the influence of the area occupied by the central feed of the bottom PRMS. Finally, using Equations (2) and (3), the bi-directional radiation patterns of the FPC metasurface antenna with arbitrary transmission-phase distribution can be calculated. Based on the theoretical analysis above, the forward and backward radiation can be controlled by the transmission-phase distributions of the top and bottom PRMSs, respectively. Note that Equations (2) and (3) only present the radiation pattern of the FPC antenna. The gain feature could be obtained by normalizing them by a factor equivalent to the radiation level of an omnidirectional antenna with the same feeding power.

## 3. Bi-Directional PFC Metasurface Antenna Design

### 3.1. Unit Design

Based on the Floquet theorem, the electromagnetic characteristics of PRMS are dependent on its unit elements, so the periodic unit structure is first designed. The periodic PRMS unit consists of three metal layers and two dielectric layers. In order to reduce the profile, the layers are pressed together without space between them. The metal layers are all copper metallic surfaces with a thickness of 0.0035 mm, and the dielectric layers are Rogers 4003 dielectrics, which are made of polytetrafluoroethylene with relative permittivity and loss angle tangent of 3.38 and 0.0027, respectively. The thickness of each dielectric layer equals 1.524 mm. The dimension of the cell is 12 mm, so that the specific dimensions are 12.00 × 12.00 × 3.38 mm^3^. The simulation is carried out using CST (Version 2023) and set with unit cell boundary conditions.

**Figure 2:**
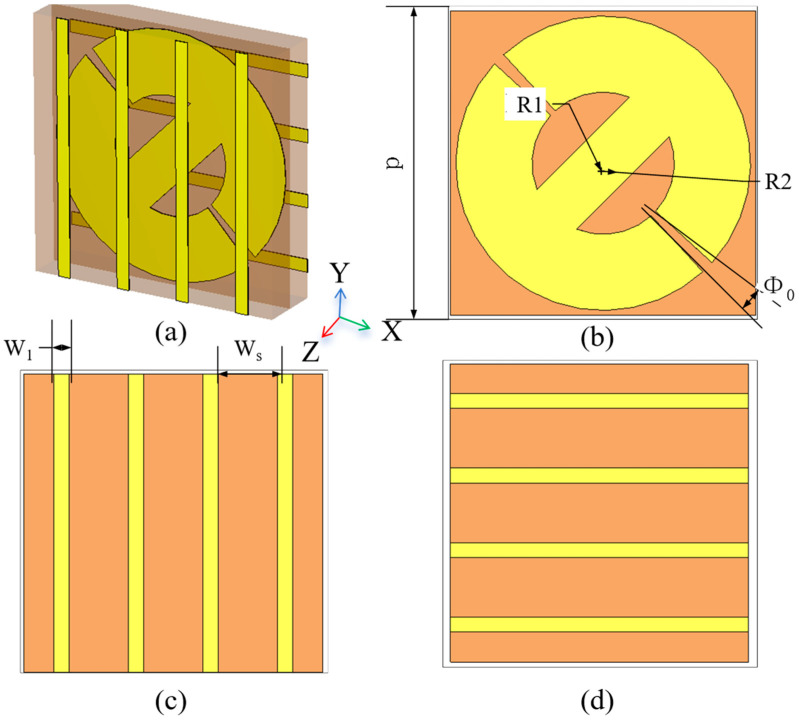
Parametric structure of the unit metal layer. (**a**) Schematic diagram of the cell; (**b**) polarization conversion layer; (**c**) top-orthogonal grid layer; and (**d**) bottom-orthogonal grid layer.

**Figure 3:**
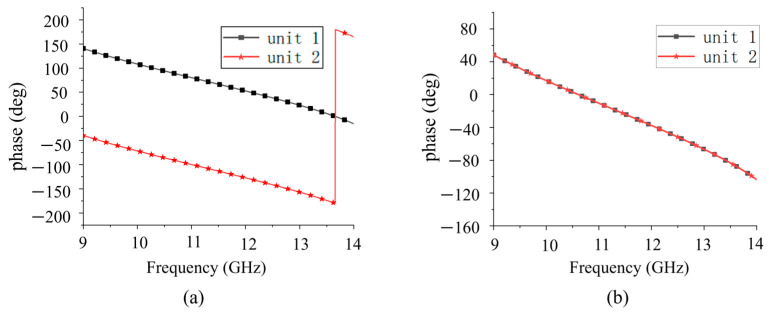
Polarization conversion layer is rotated 90° for the two units. (**a**) Transmission phase; (**b**) reflection phase.

The configuration of the PRMS substrate is shown in Figure 2a. It consists of three metal layers (top grid, intermediate trans-polarizer patch, and bottom grid) etched on two substrate layers (substrates 1 and 2). The physical cycle length *P* is set to 12 mm. The top metal grid with width *w_1_* and separation gap *w_s_* is parallel to the *y*-axis. The top and bottom metal grids in Figure 2c,d are placed in orthogonal directions, respectively, which have the same shape. Its function is opposite to the top metal grid. Two orthogonal metal grids are used to improve the transmission efficiency [29]. The middle layer acts as a polarization rotator and a phase shifter, as shown in Figure 2b. It achieves a 180° phase shift when the middle structure is rotated by 90°, defined by unit 1 and unit 2, respectively. The transmission phase of the two units is shown in Figure 3a, which exhibits a 180° phase shift at 11.5 GHz. As shown in Figure 3b, the reflection phase of the two units is the same. In the case of the electromagnetic wave incident from the bottom to the top, the device works as follows: assuming the incident wave is *x*-polarized, it is partially reflected by the bottom metallic grids at first; the transmitted wave then passes through the middle layer; the polarization of the transmitted wave is then rotated and the phase shift is controlled; and, at last, the *y*-polarized wave will transmit through the top metallic grids with a relatively low transmittance. Figure 3 also demonstrates that there is no coupling between the cross-polarized EM components, since the reflection response in *x*-polarization remains precisely constant when the transmission phase in *y*-polarization sharply shifts from 0 to π (i.e., the transmission wave is reversed). Through this design, the phase shift in the reflected *x*-polarized wave and the transmitted *y*-polarized wave can be manipulated independently.

**Figure 4:**
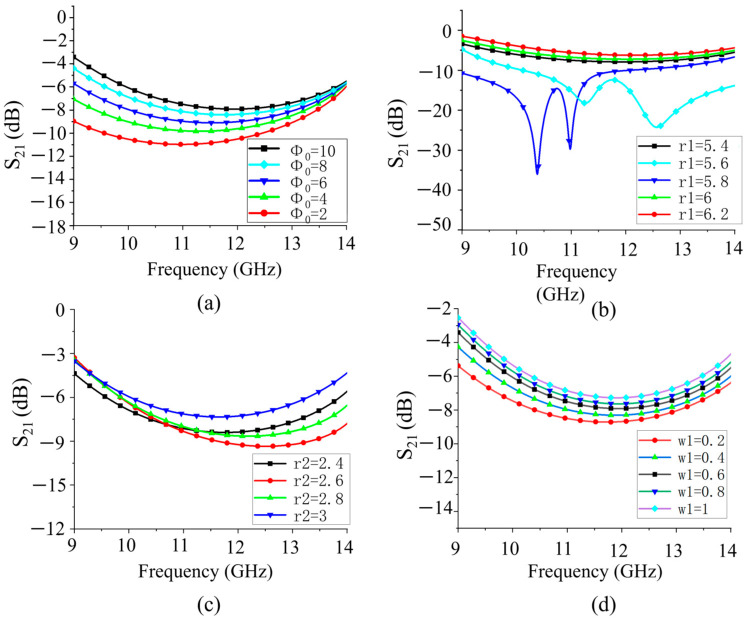
Effect of PRMS cell parameters on S_21_ (**a**) polarization conversion layer opening angle; (**b**) polarization conversion layer outer circle radius r_1_; (**c**) polarization conversion layer inner circle radius r_2_; and (**d**) metallic grid width w_1_.

Figure 4a shows the transmittance when the opening angle (Φ_0_) of the polarization conversion layer increases from 2° to 10°, which indicates that the transmittance will increase when the opening angle goes up. This is because a larger opening angle provides a larger electromagnetic wave propagation channel. Figure 4b shows the transmittance with the increase in the outer radius r_1_ of the polarization conversion layer from 5.4 mm to 6.2 mm. This indicates that the increase in the outer radius may lead to an increase in the reflection, which may reduce the transmittance. Figure 4c demonstrates the transmittance when the inner radius r_2_ of the polarization conversion layer increases from 2.8 mm to 3 mm. The increase in the inner radius may result in reduced internal reflection so that it can improve the transmittance. This phenomenon shows that the inner radius is a key factor for controlling the transmittance. In Figure 4c, it is also observed that the transmittance will decrease when the width of the metal grating (w_1_) decreases. The width of the metallic grating affects the electromagnetic wave blockage and electromagnetic field distribution. A wider metal grating may facilitate more electromagnetic wave transmission, thus increasing the transmittance. Based on the analysis above, the parameters of the unit are determined as follows: *p* = 12 mm, *w*_1_ = 0.6 mm, *h* = 1.524 mm, *r*_1_ = 5.8 mm, *r*_2_ = 2.8 mm, and *Φ*_0_ = 5°. A scattering matrix (using 11.5 GHz as an example) can be given to better characterize the transmission/reflection features in the *x*-polarized incidence case,

(4a) $$S_{1}=\left[\begin{array}{cc} t_{xx}^{1} & t_{yx}^{1}\\ r_{xx}^{1} & r_{yx}^{1} \end{array}\right]=\left[\begin{array}{cc} 0 & 0.35\\ 0.93 & 0 \end{array}\right],$$

(4b) $$S_{2}=\left[\begin{array}{cc} t_{xx}^{2} & t_{yx}^{2}\\ r_{xx}^{2} & r_{yx}^{2} \end{array}\right]=\left[\begin{array}{cc} 0 & -0.35\\ 0.93 & 0 \end{array}\right],$$

in which *S*_1_ and *S*_2_ denote the scattering matrices of unit cells 1 and 2, respectively. The transmission and reflection magnitudes are denoted by *t* and *r*, respectively, with the superscript representing the unit cell number and the subscript representing the outgoing and incident polarization. Take $t_{yx}^{1}$ as example, it represents the *y*-polarized transmission magnitude in the *x*-polarized incidence case of unit 1.

### 3.2. Feeding Antenna Design

**Figure 5:**
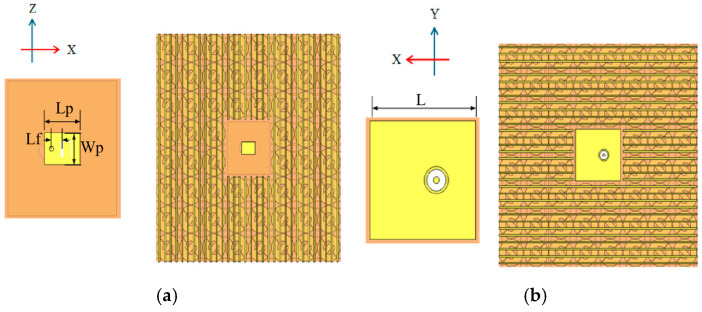
Schematic structure of microstrip patch antenna: (**a**) top view of the feeder; (**b**) bottom view of the feeder.

**Figure 6:**
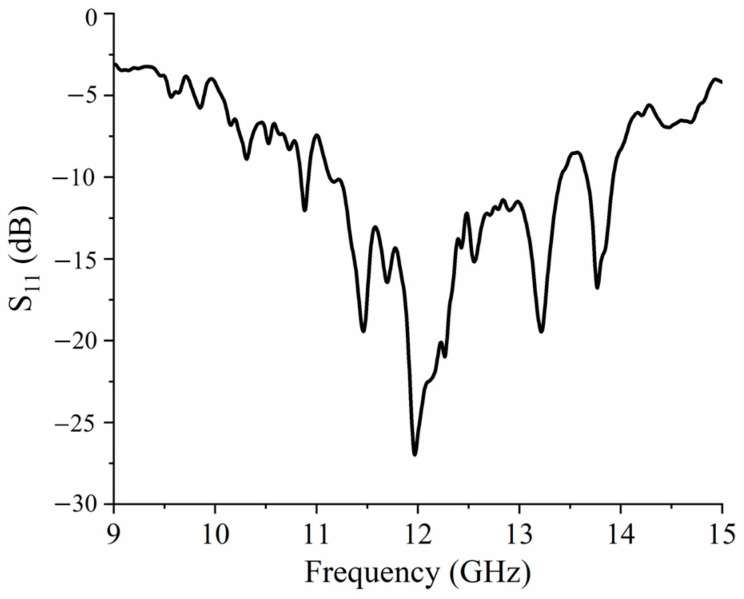
Simulation of S_11_ of feeder.

The performance of the feed antenna is crucial for the whole FPC antenna system. In this work, a rectangular patch antenna with SMA back-feed is used. This patch antenna is centered on the bottom PRMS, as shown in Figure 5. Its substrate is also the Rogers 4003. The dimension of metallic ground plane is 22 mm × 22 mm. After optimization, the dimensions of the patch are finally set to *Lp* = 7.2 mm and *Wp* = 5.2 mm, and the offset from the feeding point to the patch center equals 2.8 mm. The thickness of the antenna is 3.8 mm. The area of the feed structure is 24 mm × 24 mm, which occupies two MS cells. According to the simulation results in Figure 6, the return loss of the antenna port is less than −10 dB in the range of 10.8 GHz to 13.5 GHz, which exhibits good impedance matching when it is integrated into the FPC structure in free space. Note that the structure under simulation here only includes the bottom PRMS and feed antenna.

### 3.3. Design of the Bi-Directional FPC Metasurface Antenna

#### 3.3.1. Antenna 1 with Dual-Beam Radiation

**Figure 7:**
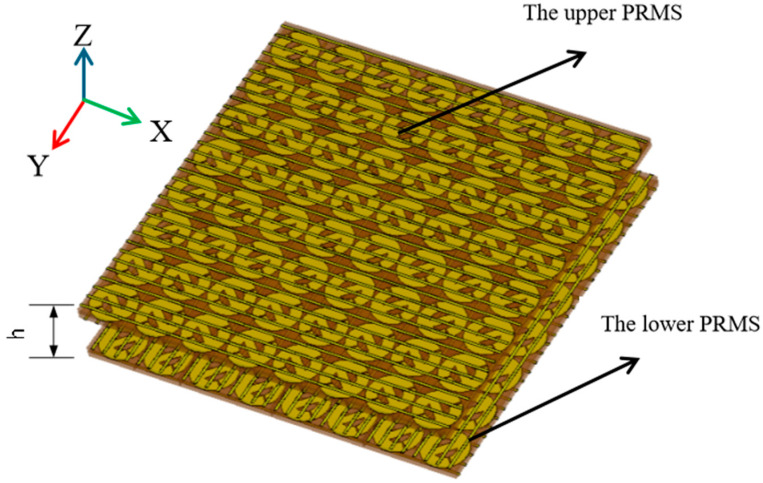
Three-dimensional schematic of the proposed bi-directional radiating FPC metasurface antenna.

Two examples of bi-directional radiation based on the FPC antenna proposed above are presented. A bi-directional FPC-MS antenna with dual-beam radiation is first designed, defined as Antenna 1. The two beams radiate forward and backward, respectively. The 3D model of Antenna 1 is shown in Figure 7, which consists of two layers of PRMS. The bottom PRMS is equipped with a feed patch antenna at its center. The feeding patch antenna is polarized along the *x*-axis, because the top and bottom grids exhibit high reflectivity for *x*-polarization. The bottom PRMS has the dimensions of 96 mm × 96 mm, with 60-unit cells. The top PRMS has the same dimensions, however, with 64 cells since there is no feeding antenna. The cavity height of the antenna equals 10.5 mm, which is based on Equation (2) and further optimized through full-wave simulations.

**Figure 8:**
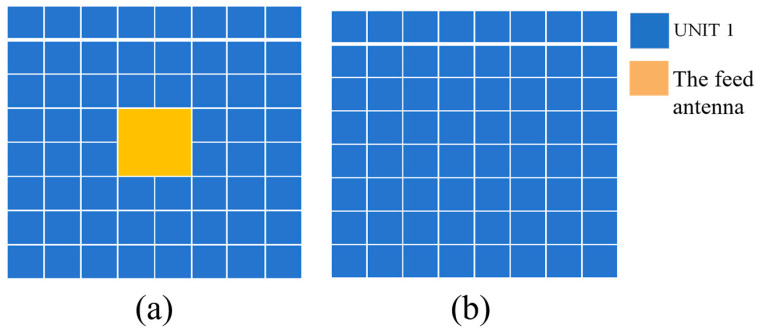
Basic unit distribution of the two PRMS layers of Antenna 1. (**a**) Unit distribution of the bottom PRMS layer (yellow filled area for the feed antenna); (**b**) unit distribution of the top PRMS layer.

The arrangement of Antenna 1 (bottom layer) is shown in Figure 8. The cell on the top and bottom PRMS is the same, employing unit 1 or 2. Herein, we employed unit 1. The arrangement of the top and bottom MSs is also the same. In Figure 8, the yellow part signifies the feed antenna.

**Figure 9:**
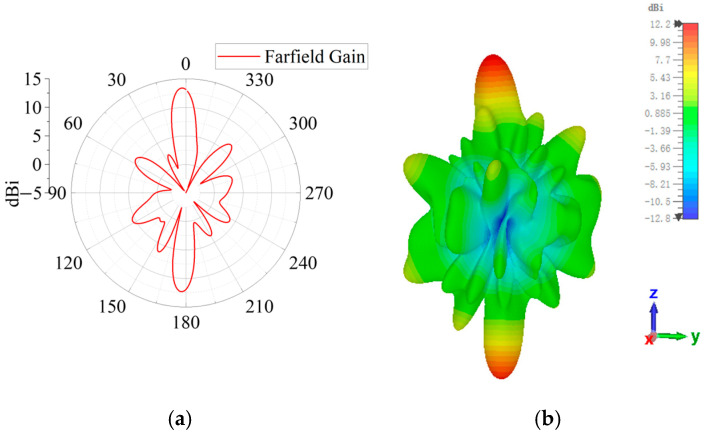
Anterior and posterior single-beam far-field radiation patterns. (**a**) Two-dimensional radiation pattern on the H-plane; (**b**) three-dimensional radiation pattern.

The simulated 2D and 3D radiation patterns are shown in Figure 9a and Figure 9b, respectively. The antenna has two radiation beams, with the two beams radiating forward and backward, respectively. The forward beam radiates in the 2° direction with a peak gain of 13.4 dBi, while the backward beam radiates in the 177° direction with a peak gain of 12.3 dBi. In this case, the dual-beam radiation is obtained by the FPC-MS antenna. In addition, because of the introduction of the polarization conversion, the polarization of two radiation beams is transformed into the cross-polarization state. This polarization conversion structure not only enhances the directivity of the antenna but also improves its overall radiation efficiency. The simulated radiation efficiency equals 83.1%.

#### 3.3.2. Antenna 2 with Tri-Beam Radiation

**Figure 10:**
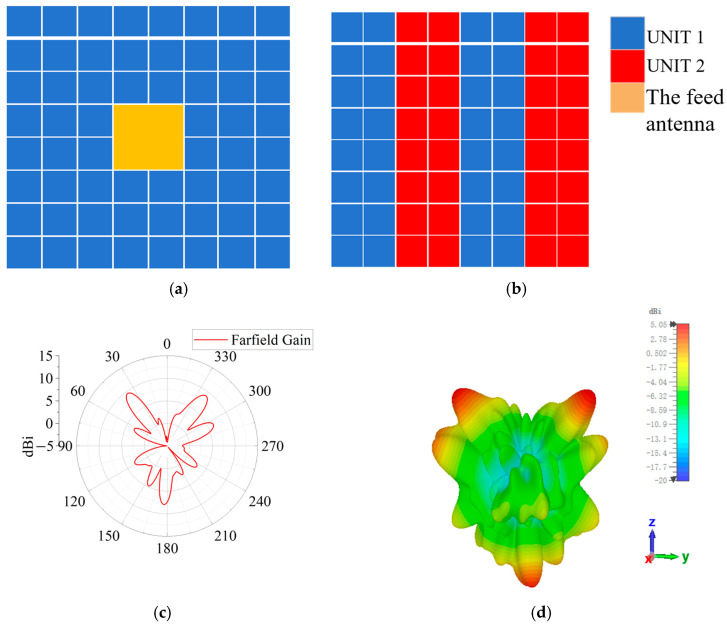
Basic unit distribution of the two PRMS layers of Antenna 2. (**a**) Unit distribution of the bottom PRMS layer (yellow filled area for the feed antenna); (**b**) unit distribution of the top PRMS layer; (**c**) 2D radiation patterns on the H-plane; and (**d**) 3D radiation pattern.

In the proposed method, the polarization characteristic is included into the FPC-MSs design, allowing the phase of *x*-polarized reflection and *y*-polarized transmission to be modulated independently. This enables the forward and backward radiations to be conveniently controlled. Therefore, another verification prototype is a tri-beam radiation antenna based on the proposed FPC method. Antenna 2 is redesigned from Antenna 1 by modifying the top layer while retaining the bottom PRMS design of Antenna 1, thereby achieving dual-beam forward radiation. In the top PRMS, two units with a 180° transmission-phase difference are used to realize this radiation characteristic, as shown in Figure 10a,b. The arrangement shown in Figure 10a is still used for the bottom layer. Based on Equations (2) and (3), the antenna effectively achieves forward dual-beam radiation and backward single-beam radiation. By redesigning the top PRMS, Antenna 2 was constructed and verified by full-wave simulation. The simulation results of the radiation pattern are shown in Figure 10c. Antenna 2 obtains a dual-beam forward and single-beam backward radiation. The forward beams fire to 37° and 322° with a gain of 9.53 dBi and 9.3 dBi, respectively. The backward single-beam fires to 178° with a gain of 7.8 dBi. The simulated radiation efficiency equals 79.3%.

## 4. Fabrication and Measurement

**Figure 11:**
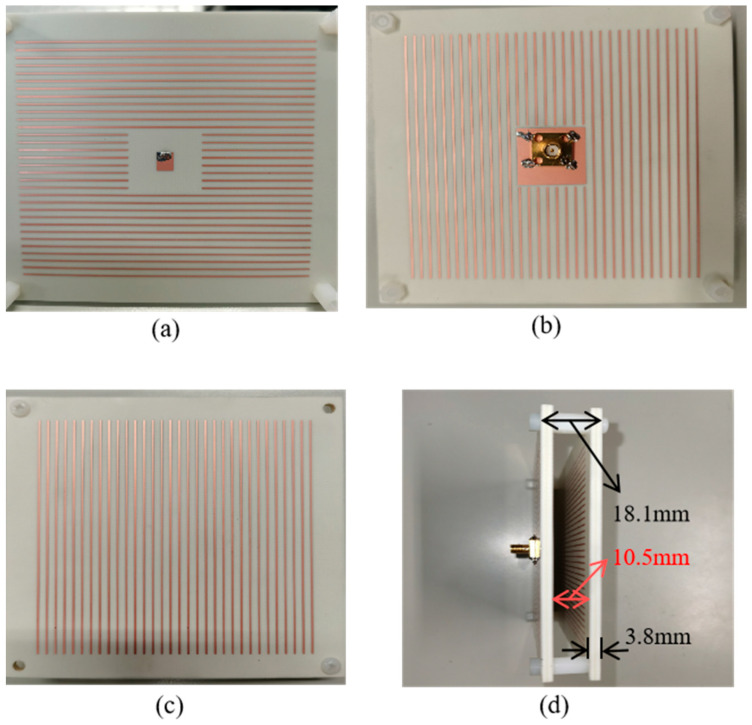
Fabricated metasurface antenna. (**a**) Front view of the feed antenna; (**b**) rear view of the feed antenna; (**c**) top layer of the integrated antenna; and (**d**) side view of the integrated antenna.

**Figure 12:**
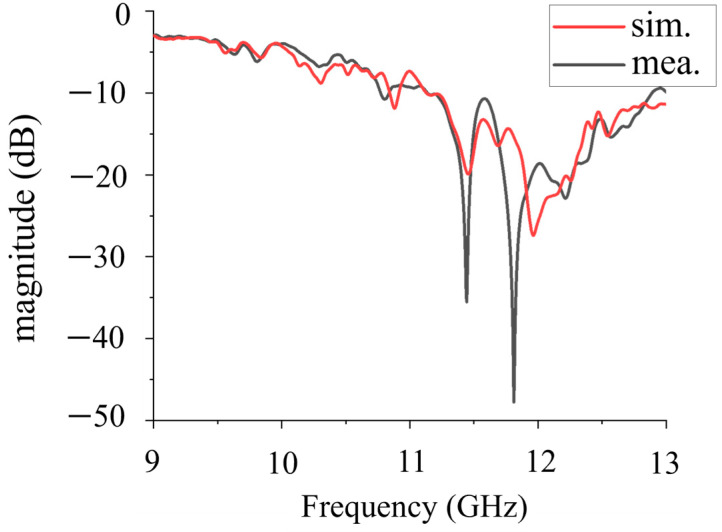
Comparison of feeder S_11_ simulation and measurement.

To further verify the proposed bi-directional FPC-MS antenna, Antenna 1 is fabricated and measured at last. The photograph of the antenna is shown in Figure 11. The antenna sample has dimensions of 106 mm × 106 mm. The thickness of each layer of PRMS is 3.8 mm. The distance between the top and bottom layers of PRMS is 10.5 mm, which is maintained by four nylon support posts. The antenna has a total profile height of 18.1 mm, around 0.69 wavelength at 11.5 GHz. First, S_11_ of the fabricated Antenna 1 was measured using a vector network analyzer (VNA Keysight N9918A (Keysight Technologies, Santa Rosa, CA, USA)). The simulated and measured results are shown in Figure 12. It shows that the measured results are in general agreement with the simulated results despite some deviations caused by the fabrication and assembly process. The reflection coefficient of the antenna sample is below −10 dB, and it shows good impedance matching in the 10.8–13 GHz band.

**Figure 13:**
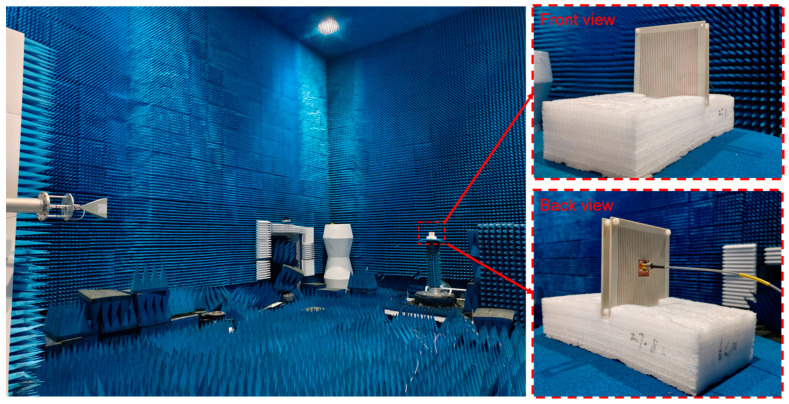
Test field for radiation direction map measurements.

**Figure 14:**
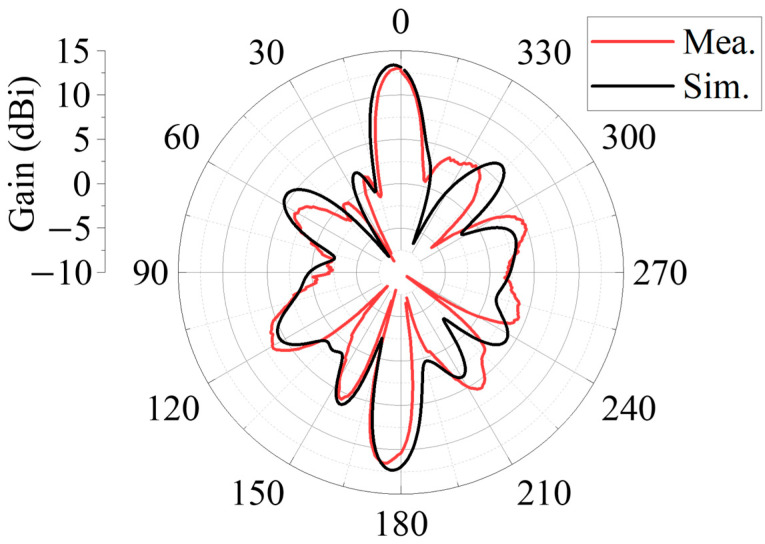
Comparison of measurement and simulation 2D radiation pattern.

Radiation performance measurement of the antenna was performed in a microwave darkroom to ensure an accurate measurement. As shown in Figure 13, the measurement system consists of the fabricated Antenna 1, vector network analyzer, a standard horn antenna covering the frequency band from 8 GHz to 12.5 GHz, and a precision rotating platform for placing the antenna prototype. The test was conducted at an operating frequency of 11.5 GHz. The co-polarized radiation pattern for Antenna 1 in the *y*-polarization was measured, and the radiation pattern result is displayed in Figure 14. The measurements are in general agreement with the simulated data. The measured data show that the forward beam is directed at 2° with a gain of 13.1 dBi. The measured gain is roughly the same as the simulated value. The backward single beam points to 175°, and its gain is approximately 11.2 dBi, which is 1.1 dB lower than the simulated value. This discrepancy is mainly because of the influence of the test cable, which is connected to the back-fed antenna on the back PRMS. The cable is positioned between the transmitter and receiver antenna, which may block some backward radiation, further decreasing the gain. The outermost layer of FPC-MS is a metallic grid in the *x*-direction, indicating that the *y*-polarized radiation cannot be emitted by the FPC; hence, the cross-polarized radiation was not measured.

In addition, the FPC antenna has a sidelobe, as listed in Table 1.

It is relatively high for two reasons. First, the electromagnetic waves in FPC do not completely radiate from the MSs, with some waves leaking from the antenna sides. Second, the transmission phase of the MS element varies with the indecent angle, resulting in slightly different phases at the center and near the edge of FPC-MS. The sidelobe caused by them can be reduced by increasing the MS aperture and finely adjusting the transmission phase in the oblique incidence cases, respectively. It should be noted that, in the measurement, (i) a millimeter-wave cable is used as the FPC antenna’s accompanying cable because, firstly, its small diameter can reduce its shielding effect of the backward radiation, and secondly, its low loss can reduce the gain loss caused by the cable; (ii) the two FPC MSs should be carefully adjusted to ensure that they are strictly parallel; and (iii) there must be no metallic structure near the FPC antenna under test because this could affect the test accuracy of the sidelobe and gain features.

To demonstrate the superiority of the bidirectional FPC antenna design method proposed in this paper, we performed a comparison of the key performance parameters achieved. These parameters—including the main operating frequency, profile height, beam pattern, bidirectional beam control independence, and polarization state—are summarized in Table 2. In contrast to other antenna designs, this work enables independent bi-directional multibeam control within the same frequency band while maintaining a low-profile configuration.

## 5. Conclusions

In this paper, a bi-directional radiating FPC antenna design based on a trans-polarization and partially reflecting MS is proposed. The transmission and reflection features can be controlled independently after introducing the polarization characteristics under consideration. The proposed bi-directional FPC-MS antenna is constructed by replacing the PRS layer and grounding plane in the conventional FPC antenna design with two PRMS layers with a trans-polarization function, resulting in a bi-directional radiation with forward and backward multibeam radiation independently controlled at the same frequency. For validation, two FPC metasurface antennas operating at 11.5 GHz with different bi-directional radiation patterns are designed and simulated. Antenna 1 performs a dual-beam radiation, of which each radiates in forward and backward regions, while Antenna 2 has a tri-beam radiation, with two beams in the forward region and one in the backward region. The performance, including high gain and directivity, is verified by the full-wave simulation. At last, Antenna 1 is fabricated and measured, with measurements showing consistency with simulation data. The study shows that, by manipulating polarization, the proposed FPC-MS antenna can realize bi-directional radiation with a low profile, of which the forward and backward beams can be manipulated independently. This research can be applied in MSs, antennas, communication, radars, and sensor systems.

## Figures and Tables

**Table 1:** Sidelobe feature of the proposed FPC antenna.

	Simulation	Measurement
Forward radiation (right space)	−7.6 dB	−8.7 dB
Forward radiation (right space)	−8.3 dB	−9.7 dB
Backward radiation (right space)	−8.6 dB	−6.0 dB
Backward radiation (left space)	−6.5 dB	−5.8 dB

**Table 2:** Comparison with some reported bi-directional radiation antennas.

Ref.	Major Operating Frequency (GHz)	Profile Height (λ)	Peak Gain (dBi)	HPBW (°)	Beam Shape	Independent Control of Bi-Directional Radiation	Polarization State for Bi-Directional Radiation
[9]	9.3	>1.18	18.5	15	Single beam	Yes	Orthogonal
[11]	1.65–2.2	0.74	8	/	Single beam	No	Same state
[14]	TA at 11; RA at 17	2.56 (11 GHz); 4 (17 GHz)	21.3 (11 GHz); 23.8 (17 GHz)	16.8 (11 GHz); 9.6 (17 GHz)	Single beam	Yes	Orthogonal
[27]	30	4	17.9	/	Multibeam	Yes	Same state
[28]	10.4	0.81	8.6	/	Multibeam	Yes	Same state
This work	11.5	0.69	13.02	13.8	Multibeam	Yes	Orthogonal

## Data Availability

The original contributions presented in this study are included in the article. Further inquiries can be directed to the corresponding authors.
